# Bleeding Risk Assessment and Management Strategies for Elective Surgery and Invasive Procedures: A Systematic Review

**DOI:** 10.7759/cureus.108286

**Published:** 2026-05-05

**Authors:** Hasan Alsagga, Mohammed I Bassiony, Osman M. B. Eladani, Bara Elhadi Elmana, Zinat Alagha, Yumna Suliman, Hatim Ahmed, Ahmed Elsir Mokhtar, Asim Ahmed

**Affiliations:** 1 General Medicine, Istanbul Medeniyet University Hospital, Istanbul, TUR; 2 Pediatric Surgery, Al Galaa Teaching Hospital, Cairo, EGY; 3 General Surgery, Menoufiya University, Shebeen El-Kom, EGY; 4 Anatomy, University of Science and Technology, Khartoum, SDN; 5 Surgery, National Ribat University, Khartoum, SDN; 6 General Medicine, Faculty of Medicine, University of Gezira, Wad Madani, SDN; 7 General Medicine, Islamic University of Gaza, Gaza, PSE; 8 Medicine and Surgery, University of Medical Sciences and Technology, Khartoum, SDN; 9 Medicine and Surgery, Al Moraya Experts Medical Complex, Jazan, SAU; 10 Anatomy, Najran University, Najran, SAU; 11 Medicine and Surgery, University of Gezira, Wad Madani, SDN

**Keywords:** bleeding disorders, bleeding risk assessment, hemostatic management, invasive procedures, perioperative bleeding

## Abstract

Patients with suspected or confirmed bleeding disorders present a distinct challenge during elective surgery and invasive procedures, as hemostatic abnormalities are heterogeneous and evidence to guide risk stratification and management remains limited, especially when routine laboratory testing is inconclusive. This systematic review aimed to synthesize available evidence on bleeding risk assessment approaches, perioperative bleeding outcomes, and hemostatic management strategies in individuals with established inherited bleeding disorders, as well as less clearly defined phenotypes such as low von Willebrand factor levels and bleeding disorders of unknown cause undergoing elective surgery or invasive procedures. We searched major electronic databases and supplementary sources for studies reporting perioperative or periprocedural bleeding outcomes and preventive or therapeutic hemostatic interventions. Due to substantial heterogeneity in study design, populations, procedures, outcome definitions, and management approaches, findings were synthesized narratively. Overall, the evidence suggests that a structured bleeding history is central to perioperative risk evaluation and may be more informative than routine coagulation screening for predicting bleeding complications, while bleeding assessment tools and laboratory parameters are inconsistently incorporated into decision-making. Hemostatic strategies, including antifibrinolytics, desmopressin, factor concentrates, and blood products, varied widely across studies and were often guided by expert judgment rather than standardized pathways. The evidence base was limited by retrospective designs, small sample sizes, and inconsistent reporting of outcomes. These findings highlight the need for prospective studies and standardized perioperative assessment and management pathways to improve procedural safety while avoiding unnecessary interventions.

## Introduction and background

Patients with suspected or confirmed bleeding disorders present a distinct perioperative challenge because even limited procedural trauma can lead to clinically significant bleeding, rescue hemostatic therapy, transfusion, reintervention, or prolonged length of stay, particularly when the bleeding phenotype is underestimated or when planning is not individualized [1]. Comparisons of perioperative outcomes across studies are further complicated by variation in how bleeding is defined, including differences in the definition of major bleeding, which limit direct comparison of reported outcomes [2].

For common inherited disorders such as von Willebrand disease, guideline-based approaches recommend stepwise diagnosis and individualized periprocedural planning based on disease subtype, baseline factor levels, expected response to desmopressin, and anticipated procedural risk [3]. Contemporary practice therefore emphasizes structured preoperative evaluation that integrates a targeted bleeding history, phenotype-driven laboratory testing, and procedure-specific risk stratification rather than reliance on routine screening tests alone.

Bleeding history assessment has evolved from informal questioning to standardized bleeding assessment tools designed to improve risk classification, harmonize referral thresholds, and reduce unnecessary testing while identifying patients who may require escalation of prophylaxis. The International Society on Thrombosis and Haemostasis Bleeding Assessment Tool (ISTH BAT) provides a standardized framework for quantifying bleeding symptoms, but its predictive performance varies by setting and patient selection [4]. Adjunctive antifibrinolytic therapy, particularly tranexamic acid, is commonly used in perioperative bleeding management and has supportive evidence across bleeding and surgical contexts, although its integration into clinical pathways remains inconsistent [5,6]. Normal ranges and reference values for bleeding scores have been described to support interpretation in both adult and pediatric populations, but score-based risk prediction remains context-dependent [7]. In addition, inconsistent classification of clinically relevant non-major bleeding can obscure interpretation when outcomes are pooled or compared [8].

Broader reviews of von Willebrand disease highlight the diversity of phenotypes and treatment approaches, reinforcing the need for individualized procedural planning [9]. Hemophilia guidelines similarly emphasize multidisciplinary perioperative care with structured replacement plans, adjunctive antifibrinolytics when appropriate, and monitoring in higher-risk settings [10]. Low von Willebrand factor represents an important intermediate phenotype with distinct diagnostic and management considerations, and periprocedural planning often requires careful integration of clinical phenotype and laboratory findings [11]. Recent guideline updates have provided more standardized approaches to diagnosis and management; however, practice variability persists, particularly when laboratory findings are borderline or procedures are heterogeneous [12,13].

Despite these advances, patients labeled as having a bleeding disorder of unknown cause remain particularly difficult to manage. The underlying mechanisms are often poorly defined, responses to prophylaxis vary, and the evidence base remains limited by small sample sizes and inconsistent outcome reporting. Recent efforts to standardize terminology and definitions in bleeding disorders of unknown cause underscore the need for prospective phenotyping, consistent endpoints, and pragmatic evaluation of periprocedural management pathways [14].

This gap supports the need for a focused systematic review of perioperative bleeding risk assessment strategies and periprocedural hemostatic management approaches in patients with suspected or confirmed bleeding disorders undergoing elective surgery or invasive procedures. The review aims to assess how these approaches relate to clinically significant bleeding and related perioperative outcomes across a broad range of bleeding phenotypes and procedural contexts.

Research question

In patients with suspected or confirmed bleeding disorders undergoing elective surgery or invasive procedures, how do preoperative bleeding risk assessment strategies and periprocedural hemostatic management approaches affect clinically significant bleeding and related perioperative outcomes?

Objectives

The primary objective of this systematic review was to evaluate evidence on preoperative bleeding risk assessment strategies used in patients with suspected or confirmed bleeding disorders undergoing elective surgery or invasive procedures.

Secondary objectives were to summarize periprocedural hemostatic management approaches and describe their reported associations with clinically significant bleeding and related outcomes, including transfusion, rescue or unplanned hemostatic therapy, reintervention, procedure delay or cancellation, length of hospital stay, and adverse events such as thrombosis. A further objective was to describe the strengths and limitations of the available evidence based on study design and risk of bias.

## Review

Methods

Protocol and Reporting Standards

This systematic review was planned and reported in accordance with the Preferred Reporting Items for Systematic Reviews and Meta-Analyses (PRISMA) 2020 statement to ensure transparent reporting of the review process, including literature search, study selection, eligibility assessment, and synthesis methods [15]. Eligibility criteria were prespecified using the Population, Intervention, Comparator, Outcomes, and Study Design (PICOS) framework to promote consistency during screening and data extraction [16]. The prespecified PICOS eligibility criteria are summarized below (see Table 1).

**Table 1:** PICOS eligibility criteria BAT, bleeding assessment tool; CRNMB, clinically relevant nonmajor bleeding; LOS, length of stay; RCT, randomized controlled trial; VWD, von Willebrand disease; VWF, von Willebrand factor; PICOS, Population, Intervention, Comparator, Outcomes, and Study Design

PICOS element	Eligibility definition
Population (P)	Patients of any age with suspected or confirmed bleeding disorders or tendencies, including: VWD, low VWF, hemophilia, rare factor deficiencies, platelet disorders, and bleeding disorders of unknown cause or unclassified, undergoing elective surgery or invasive procedures.
Intervention or Exposure (I/E)	Preoperative bleeding risk assessment strategies: (e.g., BATs, targeted laboratory evaluation, functional assays) and periprocedural hemostatic management approaches (e.g., desmopressin, antifibrinolytics, factor or VWF concentrates, platelet support, and local hemostatic measures).
Comparator (C)	Alternative assessment strategies, different prophylactic approaches, prophylaxis versus no prophylaxis, standard care versus protocol-driven or specialist-led care, or comparator groups defined within the original study.
Outcomes (O)	Clinically significant bleeding (major bleeding and/or CRNMB), transfusion, rescue or unplanned hemostatic therapy, reintervention/return to the operating room, procedure delay/cancellation, LOS, and adverse events including thrombosis.
Study Design (S)	RCTs, prospective or retrospective cohort studies, registry studies, comparative observational studies, and case series with extractable periprocedural outcomes.

Information Sources and Search Strategy

A comprehensive literature search was conducted in PubMed (MEDLINE), Embase, Scopus, and Web of Science Core Collection, with searches conducted up to early 2025 and without language restrictions. Supplementary searching included Google Scholar and backward citation searching of reference lists from included studies and relevant review articles. For Google Scholar, the first 200 results sorted by relevance were screened as a supplementary search step. The search strategy combined controlled vocabulary and free-text terms across three concept groups: (1) suspected or confirmed bleeding disorders, including von Willebrand disease, low von Willebrand factor, hemophilia, rare factor deficiencies, platelet function disorders, and bleeding disorder of unknown cause or unclassified bleeding; (2) elective surgery or invasive procedures, including endoscopy or colonoscopy, dental or oral surgery, and obstetric delivery where applicable; and (3) perioperative bleeding outcomes and hemostatic assessment or management, including bleeding assessment tools, desmopressin, antifibrinolytics such as tranexamic acid, factor or von Willebrand factor concentrates, and transfusion. Full database-specific search strategies are provided in Appendix A.

Protocol Registration

This review was not prospectively registered in PROSPERO or any other public review registry.

Study Selection Process

All identified records were exported into reference management software, where duplicate records were removed before screening. Two reviewers independently screened titles and abstracts against the prespecified eligibility criteria. Full texts of potentially relevant articles were then independently assessed by the same reviewers for final inclusion. Disagreements were resolved through discussion and consensus, with adjudication by a third reviewer when required. The study selection process was documented using a PRISMA flow diagram [15].

Data Extraction

A standardized data extraction form was developed a priori and refined before full use. Two reviewers independently extracted study-level data, including author and year, country or setting, study design, bleeding phenotype and severity where reported, procedure category, preoperative bleeding assessment strategies, periprocedural hemostatic management approaches, outcome definitions, bleeding outcomes, resource utilization outcomes, and reported adverse events, including thrombotic complications. When effect estimates were not reported, extractable procedure-level bleeding frequencies were recorded where available.

Risk of Bias Assessment

Risk of bias was assessed independently by two reviewers at the study level, with judgments recorded for each included study. Randomized controlled trials were evaluated using the Cochrane Risk of Bias 2 tool. Nonrandomized comparative studies were assessed using a structured observational risk-of-bias instrument appropriate to the study design, such as the Newcastle-Ottawa Scale or ROBINS-I. Case series were appraised using a structured critical appraisal checklist focusing on participant selection, outcome ascertainment, and completeness of follow-up. Disagreements were resolved by consensus.

Outcome Definitions

Clinically significant bleeding was defined as major bleeding and clinically relevant nonmajor bleeding, as reported by each included study. When studies used standardized definitions, we extracted and reported them, including commonly used ISTH definitions for major bleeding and clinically relevant nonmajor bleeding [2,8].

Data Synthesis

Given the anticipated heterogeneity in bleeding phenotypes, procedure types, hemostatic management strategies, and outcome definitions, a quantitative meta-analysis was not planned. Findings were synthesized narratively and grouped according to the prespecified objectives: preoperative bleeding risk assessment strategies; periprocedural hemostatic management approaches; and clinically significant bleeding and resource outcomes, stratified by procedure category. Where studies reported comparable outcome definitions, results were summarized descriptively and interpreted in the context of study design, clinical setting, and assessed risk of bias.

Results

Study Selection

The literature search identified 560 records through electronic database searching, with no additional records identified through registers or other sources. After removal of 110 duplicates, 450 unique records remained for title and abstract screening.

During screening, 350 records were excluded because they did not meet the predefined eligibility criteria. The remaining 100 reports were sought for full-text retrieval. Of these, five reports could not be retrieved, leaving 95 full-text articles for eligibility assessment.

**Figure 1:**
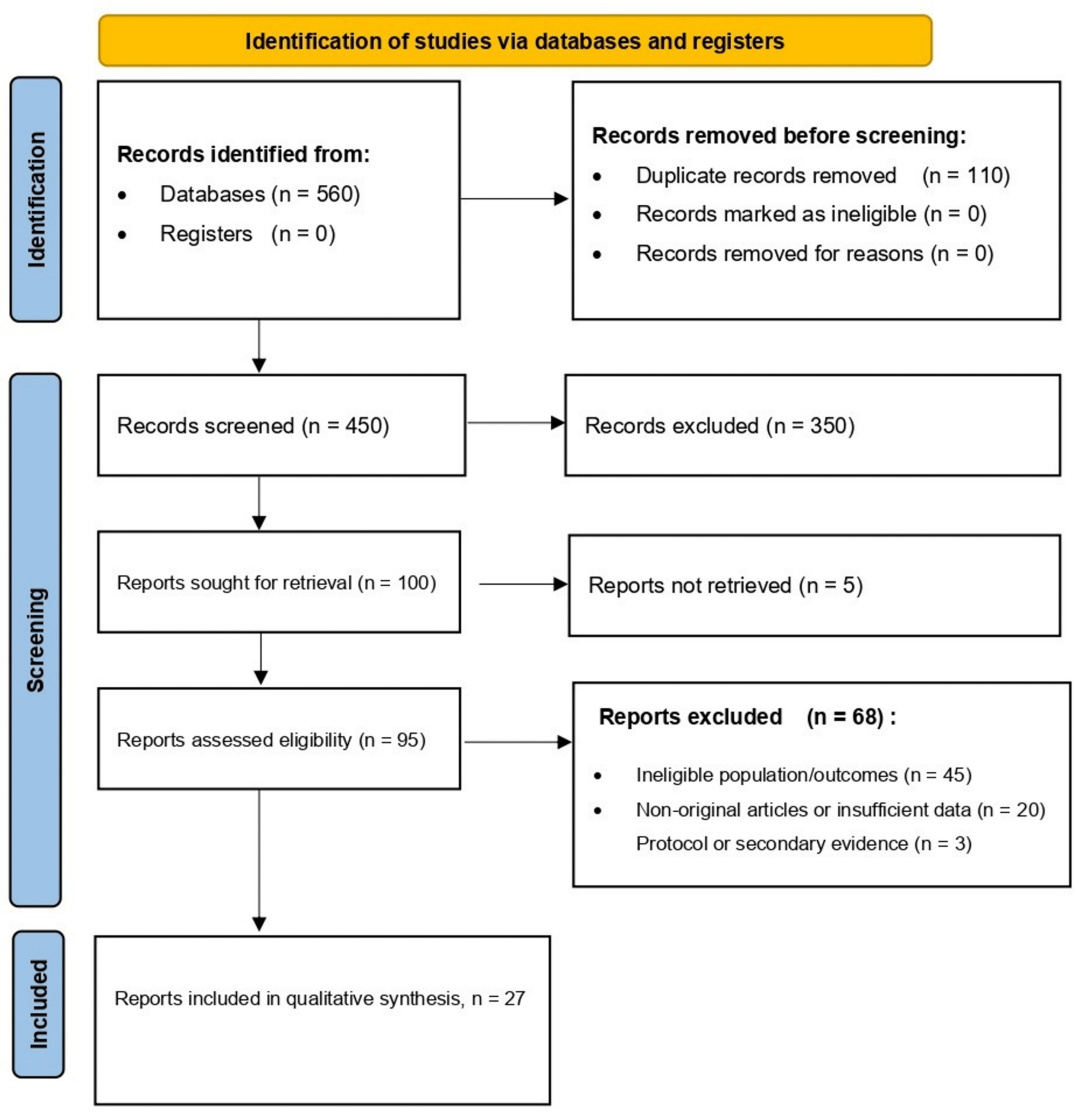
PRISMA 2020 flow diagram of study selection Flow diagram summarizing the identification, screening, eligibility assessment, and inclusion of studies in the systematic review, resulting in 27 studies included in the qualitative synthesis. PRISMA, Preferred Reporting Items for Systematic Reviews and Meta-Analyses

Following full-text review, 68 reports were excluded for specific reasons, including ineligible populations or outcomes, nonoriginal articles or studies with insufficient clinical data, and protocols or secondary evidence such as review articles. A total of 27 studies met the inclusion criteria and were included in the qualitative synthesis. The study selection process is summarized in Figure 1.

Characteristics of Included Studies

Across the included studies [17-43], the evidence base comprised prospective and retrospective cohort studies, registry analyses, randomized clinical trials, and case series, covering a broad range of bleeding phenotypes and procedural contexts. Included populations encompassed von Willebrand disease, low von Willebrand factor, hemophilia, rare factor deficiencies, platelet disorders, and bleeding disorders of unknown cause. Procedures included elective major surgery, gastrointestinal endoscopy and colonoscopy, oral and dental procedures, and obstetric interventions. Table 2 summarizes the population, procedural context, study design, primary focus, and key reported outcome for each included study.

**Table 2:** Characteristics of included studies (n=27) This table summarizes the clinical characteristics and management strategies of 27 studies on perioperative care for patients with bleeding disorders. BDUC or BUC, bleeding disorder or bleeding of unknown cause; DDAVP, desmopressin; FXI or FXI:C, factor XI or factor XI activity; GI, gastrointestinal; HTC, hemophilia treatment center; ISTH BAT, International Society on Thrombosis and Haemostasis Bleeding Assessment Tool; PK or TGA, pharmacokinetics or thrombin generation assay; PPH, postpartum hemorrhage; rFVIIa or rVWF, recombinant activated factor VII or recombinant von Willebrand factor; TXA, tranexamic acid; VWD or VWF, von Willebrand disease or von Willebrand factor

Study	Population	Procedural context	Design	Primary focus	Key reported outcome
Ambaglio C, 2021 [17]	Suspected bleeding disorder	Elective surgery	Prospective cohort	ISTH BAT and lab screen	Limited predictive value for bleeding
Clarke L, 2021 [18]	Inherited bleeding disorders	Elective procedures	Retrospective cohort	Guideline-based care	Low complication rates under structured care
Tomaszewski M, 2019 [19]	Bleeding disorders	GI endoscopy	Retrospective cohort	Periendoscopic care	Low bleeding with tailored prophylaxis
Azer SM, 2020 [20]	Bleeding disorders	Colonoscopy	Retrospective cohort	Prophylaxis strategy	Risk-based prophylaxis supported
Fribourg E, 2024 [21]	Inherited bleeding disorders	Oral surgery	Retrospective cohort	Oral surgical outcomes	Mostly manageable postoperative bleeding
Napolitano M, 2015 [22]	Factor VII deficiency	Surgery	Observational	Replacement safety	Surgery feasible with monitoring
Obaji S, 2016 [23]	Unclassified bleeding disorder	Mixed surgery	Retrospective review	TXA and/or DDAVP	High rate of successful hemostasis
Doherty D, 2021 [24]	Low VWF	Elective procedures	Retrospective cohort	DDAVP vs. TXA	DDAVP effective; omission increased risk
Veen CS, 2021 [25]	Bleeding of unknown cause	Surgery and delivery	Observational	Outcome burden	High surgical and obstetric bleeding risk
Rousseau F, 2024 [26]	Rare bleeding disorders	Surgery	Prospective cohort	Surgical outcomes	Contemporary safety benchmarks
Peyvandi F, 2019 [27]	Severe VWD	Elective surgery	Phase 3 trial	Recombinant VWF	Effective perioperative hemostasis
Hazendonk HC, 2018 [28]	VWD	Surgery and dental	Retrospective	Practice patterns	Need for personalized dosing
Dobrkovska A, 1998 [29]	VWD	Surgery	PK and clinical study	Humate P	High efficacy reported
de Jager NC, 2020 [30]	VWD	Elective surgery	Population PK	FVIII kinetics	Clearance influenced by surgery duration
Miesbach W, 2015 [31]	VWD	Surgery	Retrospective cohort	Hemate P	Majority of procedures uncomplicated
Lewandowski B, 2018 [32]	Mild hemophilia and VWD	Dental extraction	Clinical report	No supplementation	Select low-risk feasibility
Saulnier J, 1994 [33]	Mild/moderate disorders	Dental extraction	Clinical evaluation	DDAVP and TXA	Bleeding largely prevented
Forbes CD, 1972 [34]	Hemophilia	Dental extraction	Double-blind trial	TXA	Reduced post-extraction bleeding
Nuvvula S, 2014 [35]	Hemophilia	Dental scaling	Randomized trial	TXA mouthwash	Effective alternative to factor
Goudemand J, 2020 [36]	VWD	Surgeries including childbirth	Prospective observational (post-marketing)	FVIII-poor pdVWF concentrate real-world use	High rate of excellent/good hemostasis across surgeries; childbirth included
Gill JC, 2011 [37]	VWD	Elective surgery	Prospective multicenter (phase IV)	PK-guided dosing of VWF/FVIII (Humate-P)	Effective hemostasis in most cases; some post-op hemorrhage despite target levels
Seaman CD, 2019 [38]	VWD	Periprocedural care	Retrospective (institutional experience)	Real-world periprocedural management approach	Practical outcomes from institutional peri-procedural pathway (bleeding outcomes + management patterns)
Di Minno MN, 2018 [39]	Factor VII deficiency	Surgery	Registry study	Treatment intensity	Prior bleeding predicts need
Mariani D, 2011 [40]	Factor VII deficiency	Surgery	Prospective registry	rFVIIa dosing	Effective perioperative use
Berkowitz C, 2024 [41]	BDUC	Procedures and delivery	Retrospective cohort	Prophylaxis	Reduced surgical; persistent PPH
Rhoades R, 2023 [42]	Bleeding disorders	Major surgery	Retrospective cohort	HTC care	Low major bleeding rates
Désage S, 2022 [43]	Factor XI deficiency	Surgery	Retrospective cohort	TGA	TGA outperformed FXI:C

Preoperative Bleeding Assessment and Predictors

Across studies evaluating structured preoperative assessment, traditional screening approaches, including routine coagulation tests and bleeding assessment tools, showed limited sensitivity for detecting mild or previously unrecognized bleeding risk in unselected elective surgical populations in Ambaglio et al. [17]. In Clarke et al. [18], outcomes were generally favorable in patients with established inherited bleeding disorders managed within structured specialist pathways. Procedure-specific cohorts in gastrointestinal endoscopy and colonoscopy reported favorable outcomes when prophylaxis was tailored to procedural bleeding risk rather than applied as uniform factor replacement strategies [19,20].

In factor XI deficiency, thrombin generation assay parameters were reported to be more informative than factor XI activity alone, and impaired thrombin generation was associated with increased perioperative bleeding [43]. In factor VII deficiency, a history of prior major bleeding predicted the intensity of replacement therapy required [39,40]. In bleeding disorders of unknown cause, included studies reported higher surgical and obstetric bleeding rates than in classified bleeding disorders [25,41]. Key predictors identified across studies are summarized in Table 3.

**Table 3:** Preoperative bleeding risk assessment and predictors This table summarizes key predictors of perioperative bleeding identified across included studies. ASA, American Society of Anesthesiologists; BDUC, bleeding disorder of unknown cause; FXI, factor XI; FXI:C, factor XI activity; ISTHBAT, International Society on Thrombosis and Haemostasis Bleeding Assessment Tool; PK, pharmacokinetics; TGA, thrombin generation assay; VWD, von Willebrand disease; VWF, von Willebrand factor; PPH, postpartum hemorrhage

Domain	Evidence summary	Supporting studies
Screening tools	Limited predictive ability of ISTH BAT and routine labs in unselected populations	Ambaglio C, 2021 [17]
Functional assays	TGA superior to FXI:C in predicting surgical bleeding	Désage S, 2022 [43]
Clinical history	Prior major bleeding predicts replacement needs in FVII deficiency	Di Minno MN, 2018 [39]; Mariani G, 2011 [40]
Precision dosing	PK modeling improves VWF target attainment	de Jager NC, 2020 [30]
Unknown cause	BDUC associated with higher surgical bleeding and PPH	Veen CS, 2021 [25]; Berkowitz C, 2024 [41]

Hemostatic Management Strategies

Reported periprocedural hemostatic management strategies were generally risk-stratified across included studies. In von Willebrand disease, recombinant von Willebrand factor was reported as effective for major elective surgery, and plasma-derived concentrates were reported as safe and effective across procedures [27,29,31]. Population pharmacokinetic modeling was used to improve dosing precision in complex surgical settings [30]. In patients with low von Willebrand factor levels, desmopressin was effective for elective procedures, whereas tranexamic acid alone was often used for selected minor interventions [24]. In bleeding disorders of unknown cause, studies reported procedural success with antifibrinolytics and desmopressin, although obstetric bleeding remained a persistent challenge [23,25,41]. Practical management patterns across procedure categories are summarized in Table 4.

**Table 4:** Peri-procedural hemostatic management strategies and outcomes This table summarizes commonly reported hemostatic strategies and outcome patterns across procedural categories. ABDUC, bleeding disorder of unknown cause; DDAVP, desmopressin; HTC, hemophilia treatment center; PPH, postpartum hemorrhage; rFVIIa, recombinant activated factor VII; rVWF, recombinant von Willebrand factor; TXA, tranexamic acid; VWD, von Willebrand disease; VWF, von Willebrand factor; PPH, postpartum hemorrhage

Procedure category	Typical strategy	Outcome pattern
Major surgery	Specialist-led care	Low major bleeding rates
VWD surgery	Personalized VWF dosing	High efficacy
Low VWF	DDAVP ± TXA	Increased bleeding if prophylaxis omitted
FVII deficiency	rFVIIa	Effective when phenotype-guided
GI endoscopy	Selective prophylaxis	Safe with risk-based approach
Oral/dental	Antifibrinolytics	Bleeding generally manageable
BDUC obstetrics	TXA/DDAVP	Persistently high PPH

Risk of Bias Assessment

Risk of bias varied across individual studies and generally reflected study design. The randomized dental trials [34,35] were less vulnerable to selection bias, although both were limited by small sample sizes and older methodology. Prospective cohorts and registry studies [17,26,36,37,39,40] reduced selection bias through consecutive enrollment or structured data collection, but remained limited by event rates, confounding, or treatment heterogeneity. Most retrospective cohorts [18-21,24,25,28,31,38,41-43] were conducted in specialized referral centers and were more susceptible to selection and information bias. A summary of study design strengths and principal bias concerns is provided in Table 5.

**Table 5:** Summary of risk of bias across included studies Risk of bias was assessed at the study level using RoB 2 for randomized trials, structured observational tools for prospective and registry studies, and checklist-based appraisal for retrospective cohorts and case series. ROB, risk of bias

Study design	Main strengths	Principal bias concerns
Randomized trials	Blinding; controlled interventions	Small sample sizes; older methodology
Prospective cohorts	Consecutive enrollment	Limited event rates
Registries	Large sample size	Treatment heterogeneity
Retrospective cohorts	Real-world data	Selection and documentation bias

Discussion

This systematic review found that perioperative and periprocedural outcomes in patients with confirmed inherited bleeding disorders were generally favorable when care was delivered in experienced settings using structured pathways. However, evidence for accurate risk prediction and standardized management remained limited by heterogeneous phenotypes, variable procedural risk, and inconsistent definitions of bleeding endpoints. Across the included literature, the most consistent signal was that outcomes depended as much on phenotype characterization and procedural context as on any single screening test or prophylaxis agent. These findings support the need for integrated risk assessment models rather than reliance on routine laboratory screening alone.

Evidence from unselected elective surgical populations suggests that commonly used screening approaches may be poorly calibrated for identifying clinically meaningful occult bleeding risk. In Ambaglio et al. [17], abnormal screening by ISTH BAT and/or routine laboratory testing was uncommon, and major bleeding did not concentrate within the screen-positive subgroup, indicating limited discriminatory performance of conventional screening in low-pretest-probability settings. In contrast, in cohorts of patients with known inherited bleeding disorders undergoing elective procedures, outcomes were generally reassuring under structured care pathways. Clarke et al. [18] reported low complication rates despite deviations from published guidance, which may reflect clinician-led risk adaptation in real-world care rather than uniformly unsafe practice. These favorable outcomes under specialist care should not be interpreted as direct evidence of predictive accuracy. Rather, together, these findings suggest that preoperative algorithms should prioritize individualized assessment that incorporates bleeding phenotype and procedural complexity, with laboratory evaluation used to refine risk and guide therapy rather than replace clinical stratification.

Procedure-specific evidence further supported risk-matched prophylaxis rather than uniform treatment. In gastrointestinal endoscopy cohorts, bleeding outcomes were generally favorable when prophylaxis intensity aligned with procedural risk and intervention type. However, reporting was often insufficient to quantify risk across diagnostic versus therapeutic procedures or to compare standardized follow-up windows [19,20]. This limitation is clinically important because delayed bleeding can remain unascertained without consistent postprocedure surveillance. The available evidence supports prospective operationalization of endoscopy risk categories with uniform bleeding definitions and follow-up intervals to improve interpretability and reduce between-study variation.

Oral surgery and dental procedures demonstrated a distinct pattern. Bleeding events were not uncommon in some cohorts but were typically manageable with local measures and appropriately selected adjunctive systemic therapy. Fribourg et al. [21] reported postoperative bleeding events that were generally controllable in inherited bleeding disorders, highlighting that pathway reliability, patient education, and rescue capacity may influence outcomes as strongly as prophylaxis selection. In rare factor deficiencies such as factor VII deficiency, observational and registry evidence supported the feasibility of surgery under replacement strategies. Still, interpretation remains vulnerable to confounding by indication because patients perceived as higher risk are more likely to receive intensive prophylaxis [22,39,40]. Future registry reporting should stratify outcomes by baseline phenotype severity, procedure invasiveness, and prophylaxis intensity to enable more meaningful comparisons between strategies.

Among partially characterized phenotypes, including low von Willebrand factor and unclassified bleeding disorders, evidence suggested that lower-intensity prophylaxis, often antifibrinolytics with or without desmopressin, achieved hemostatic success for many procedures. However, risk was not negligible and appeared concentrated in higher-risk contexts. Obaji et al. [23] supported a stepwise approach using tranexamic acid and/or desmopressin in unclassified bleeding disorders. Similarly, Doherty et al. [24] indicated a benefit of desmopressin-centered strategies in low von Willebrand factor, with increased bleeding when hemostatic cover was withheld in selected settings. These findings support treating such phenotypes as risk-modifiable rather than low risk by default, with escalation rules linked to personal bleeding history and procedure class.

The most clinically important gap identified in this review was the persistently high bleeding burden associated with bleeding disorder of unknown cause, particularly in major surgery and obstetrics. Veen et al. [25] and Berkowitz et al. [41] described a substantial bleeding burden in this group, with prophylaxis appearing to mitigate but not eliminate adverse outcomes, and postpartum hemorrhage remaining a prominent unmet need. In contrast, outcomes in defined disorders managed in specialized centers were comparatively favorable, including major surgery cohorts in hemophilia treatment center settings [42]. This contrast supports the view that diagnostic clarity may enable targeted, mechanism-based therapy and more reliable periprocedural planning. At the same time, the biological heterogeneity of bleeding disorders of unknown cause likely limits the effectiveness of strategies extrapolated from classified disorders. Addressing this gap will require prospective studies with standardized obstetric endpoints and pragmatic evaluation of multimodal prophylaxis pathways rather than single-agent strategies.

Von Willebrand disease provided the clearest example of progress toward individualized, pharmacokinetic-informed management. Trial evidence supported the efficacy of recombinant von Willebrand factor in severe disease under protocolized conditions [27]. Observational evidence supported the effectiveness of plasma-derived concentrates in real-world specialist care [31]. Studies evaluating practice patterns and pharmacokinetic variability emphasized that interindividual differences can materially affect factor trajectories and that monitoring intensity and system capacity may be as important as product selection [28,30]. This supports interpreting product effectiveness in the context of implementation, including access to monitoring, dose adjustment, and multidisciplinary oversight.

Adjunctive antifibrinolytic therapy was consistently supported in dental contexts, aligning with mucosal bleeding biology and enabling factor-sparing approaches in appropriate patients. Older randomized evidence supported systemic tranexamic acid for reducing postextraction bleeding [34]. More recent trials have supported the use of topical antifibrinolysis during dental interventions in patients with hemophilia [35]. Desmopressin also appeared beneficial for selected dental procedures in hemostatic disorders [33]. Although these studies varied in endpoints and procedural contexts, their collective findings support structured dental pathways based on local measures and antifibrinolysis, reserving concentrates for higher-risk anatomy, severe phenotypes, or failed first-line strategies.

Finally, functional global assays emerged as a promising bridge between phenotype and laboratory prediction in settings where isolated factor activity does not reliably track bleeding. In factor XI deficiency, thrombin generation assay parameters appeared more informative than factor XI activity alone for perioperative bleeding prediction [39-43]. While availability and standardization remain barriers, this line of evidence supports further evaluation of capacity-based assays within integrated prediction models, particularly for disorders where conventional testing underperforms.

Overall, the evidence base remains constrained by predominantly retrospective designs, referral center selection, inconsistent endpoint definitions, and heterogeneous follow-up windows, limiting comparative effectiveness inference. Future research should prioritize prospective, multicenter studies that standardize definitions of major bleeding and clinically relevant nonmajor bleeding, prespecify procedure-specific follow-up windows, include explicit obstetric endpoints, and assess integrated strategies combining bleeding history, procedure class, targeted laboratory testing, and, where appropriate, pharmacokinetic-guided dosing and functional assays.

## Conclusions

Perioperative outcomes for patients with confirmed inherited bleeding disorders are generally favorable when managed in experienced centers with structured multidisciplinary pathways. However, risk prediction and standardized management remain limited by heterogeneous bleeding phenotypes, variable procedural risk, and inconsistent outcome definitions, and bleeding risk appears to be driven more by phenotype and procedural context than by any single screening test or prophylaxis agent.

Patients with bleeding disorders of unknown cause remain the most challenging group, with a higher burden of bleeding, particularly in major surgery and obstetrics, despite prophylaxis. Future research should prioritize prospective multicenter studies with standardized bleeding definitions, procedure-specific follow-up, and explicit obstetric endpoints. These studies should also evaluate integrated pathways combining structured bleeding history, procedure classification, targeted laboratory testing, and, where appropriate, pharmacokinetic-guided dosing and functional assays.

## Appendices

Appendix A 

**Table 6:** Detailed electronic search strategies for the identification of studies on inherited bleeding disorders and perioperative management BAT, Bleeding Assessment Tool; BDUC, bleeding disorder of unknown cause; DDAVP, desmopressin; rFVIIa, recombinant activated factor VII; TGA, thrombin generation assay; TXA, tranexamic acid; VWD, von Willebrand disease; VWF, von Willebrand factor

Database/platform	Search strategy/query	Notes and instructions
PubMed (MEDLINE)	("Blood Coagulation Disorders, Inherited"[Mesh] OR "Blood Coagulation Disorders"[Mesh] OR "von Willebrand Diseases"[Mesh] OR "von Willebrand Factor"[Mesh] OR "von Willebrand disease"[tiab] OR vwd[tiab] OR "low VWF"[tiab] OR "low von Willebrand factor"[tiab] OR "Hemophilia A"[Mesh] OR "Hemophilia B"[Mesh] OR hemophilia*[tiab] OR "Factor VII Deficiency"[Mesh] OR "Factor XI Deficiency"[Mesh] OR "Factor XIII Deficiency"[Mesh] OR "Fibrinogen Deficiency"[Mesh] OR "blood coagulation factor deficien*"[tiab] OR "rare bleeding disorder*"[tiab] OR "Platelet Function Disorders"[Mesh] OR "platelet function disorder*"[tiab] OR "inherited platelet disorder*"[tiab] OR "bleeding disorder of unknown cause"[tiab] OR "bleeding of unknown cause"[tiab] OR "unclassified bleeding disorder*"[tiab] OR "undetermined bleeding disorder*"[tiab] OR "suspected bleeding disorder*"[tiab]) AND ("Surgical Procedures, Operative"[Mesh] OR surgery[tiab] OR surgical[tiab] OR perioperativ*[tiab] OR periprocedur*[tiab] OR intraoperativ*[tiab] OR postoperativ*[tiab] OR "invasive procedure*"[tiab] OR endoscop*[tiab] OR colonoscop*[tiab] OR gastroscop*[tiab] OR "Dental Extraction"[Mesh] OR dental[tiab] OR "oral surgery"[tiab] OR extraction*[tiab] OR obstetric*[tiab] OR deliver*[tiab] OR childbirth[tiab] OR "Delivery, Obstetric"[Mesh] OR "Postpartum Hemorrhage"[Mesh]) AND (bleed*[tiab] OR hemorrhag*[tiab] OR haemorrhag*[tiab] OR "blood loss"[tiab] OR transfusion*[tiab] OR hemostas*[tiab] OR "hemostatic management"[tiab] OR prophylax*[tiab] OR "bleeding assessment tool*"[tiab] OR "bleeding score"[tiab] OR "ISTH BAT"[tiab] OR "bleeding history"[tiab] OR "thrombin generation"[tiab] OR "thrombin generation assay"[tiab] OR TGA[tiab] OR "Desmopressin"[Mesh] OR desmopressin[tiab] OR ddavp[tiab] OR "Tranexamic Acid"[Mesh] OR tranexamic[tiab] OR antifibrinolytic*[tiab] OR "Factor Concentrates"[Mesh] OR "blood coagulation factor concentrate*"[tiab] OR "von Willebrand factor concentrate*"[tiab] OR "recombinant factor VIIa"[tiab] OR rFVIIa[tiab] OR "platelet transfusion"[tiab] OR "Blood Transfusion"[Mesh])	Run without limits unless specified. Report any applied filters (e.g., date range, humans).
Embase (Elsevier/Ovid)	Uses Emtree terms such as 'blood coagulation disorder'/exp, 'von willebrand disease'/exp, and 'surgery'/exp. Combined with keywords using the :ti,ab,kw tag for Title, Abstract, and Keywords.	Controlled vocabulary is platform-dependent. Verify Emtree mapping in your specific interface before running.
Scopus (Elsevier)	Uses the TITLE-ABS-KEY operator to combine the three keyword clusters (Conditions, Procedures, and Outcomes).	Export the full record and abstract for the screening phase.
Web of Science	Uses the TS= (Topic) tag to search across titles, abstracts, and author keywords.	Export the full record and abstract for screening.
Google Scholar	Example: ("von Willebrand" OR hemophilia OR "low VWF" OR "bleeding disorder of unknown cause" OR "unclassified bleeding disorder") (perioperative OR surgery OR "invasive procedure" OR endoscopy OR colonoscopy OR dental OR "oral surgery" OR obstetric OR delivery)	Supplementary source. Document the screening rule (e.g., first 200 results) and the date of execution.
